# Nationwide Molecular Epidemiology of HIV‐1 in Uruguay (2007–2021): Lineage Diversity, BF1 Recombinant Complexity and Epidemiological Patterns

**DOI:** 10.1002/jia2.70157

**Published:** 2026-07-25

**Authors:** Agustina A. Podchibiakin, Rosa Flieller, Dora Ruchansky, Maria Brasesco, Maria N. Cortinas, Andrés Lizasoain, Héctor Chiparelli, Susana Cabrera, Gonzalo Bello, Ana M. Soler, Daiana Mir

**Affiliations:** ^1^ Universidad de la República – CENUR Litoral Norte, Department of Biological Sciences Genomics and Bioinformatics Unit Salto Uruguay; ^2^ Ministry of Public Health, Department of Public Health Laboratories Virology Unit Montevideo Uruguay; ^3^ Ministry of Public Health, Department of Public Health Laboratories Genomics Unit Montevideo Uruguay; ^4^ Universidad de la República – CENUR Litoral Norte, Department of Biological Sciences Molecular Virology Laboratory Salto Uruguay; ^5^ Universidad de la República, Faculty of Medicine Montevideo Uruguay; ^6^ Fiocruz, Instituto Oswaldo Cruz Arbovirus and Hemorrhagic Virus Laboratory Rio de Janeiro Brazil; ^7^ Universidad de la República – CENUR Litoral Norte, Department of Biological Sciences Human Molecular Genetics Laboratory Salto Uruguay

**Keywords:** genomic surveillance, HIV‐1, Latin America, molecular epidemiology, phylogenetics, Uruguay

## Abstract

**Introduction:**

Uruguay has one of the highest HIV incidence rates in Latin America, yet its molecular epidemiology remains underexplored. Characterizing the diversity, temporal and geographic dynamics of circulating HIV‐1 lineages is essential to detect lineage‐specific patterns across population groups and regions, inform tailored prevention and surveillance strategies, and understand cross‐border connectivity.

**Methods:**

HIV‐1 *pol* (PR/RT) genotyping data from 1268 people living with HIV, generated during 2007–2021 through nationwide routine genotyping, were analysed. Viral lineages and recombinants were classified using automated subtyping tools, maximum‐likelihood phylogenies and recombination analyses. Associations with demographic and exposure characteristics were evaluated using Z‐tests and Fisher's exact tests, and temporal trends were assessed using Pearson correlations.

**Results:**

Sixteen recognized HIV‐1 lineages were identified, including four subtypes/sub‐subtypes (B, C, A1 and F1) and twelve circulating recombinant forms (CRFs). Subtype B was the most frequent lineage (39.3%), followed by CRFs 12_BF (27.8%) and 38_BF1 (12.9%). An additional 8.5% of sequences formed a well‐supported B/F1 recombinant clade, provisionally designated BF1.UY and not assignable to any known CRF. Temporal analyses showed opposing trends, with a significant decline in subtype B and an increasing contribution of BF1 recombinants within the genotyped dataset. Subtype B was associated with men and transmission among men who have sex with men; 38_BF1 was enriched among women and people reporting injection‐related exposures; and BF1.UY predominate in reported heterosexual transmission. Geographic patterns suggested regional connectivity: 12_BF predominated in the West and the Atlantic coastal East, consistent with Argentina‐linked border and tourism‐related mobility, whereas Brazilian‐associated variants (subtype C, 31_BC and F1) were more frequent in the Northeast and East.

**Conclusions:**

In Uruguay, HIV‐1 molecular diversity was largely shaped by sustained circulation of subtype B and BF1 recombinants, which showed distinct associations with gender, transmission‐route category and geography. These lineage‐specific patterns are consistent with partially overlapping transmission networks among Uruguayan subpopulations and viral exchange across border areas. The growing contribution of BF1 recombinants within the genotyped dataset, together with geographically structured diversity in areas connected to Argentina and Brazil, highlights the need for sustained molecular surveillance integrated with epidemiological data and regional collaboration to track HIV‐1 diversification and spread.

## 1 Introduction

Over the past decade, the global HIV epidemic has shown substantial but uneven progress. In 2024, an estimated 1.3 million people (1.0−1.7 million) acquired HIV, representing a 40% decline in annual global incidence since 2010. However, this overall decline masks marked regional disparities. Latin America is one of the few regions where HIV incidence has increased, rising by 13% between 2010 and 2024 [1]. This rise unfolds within a broader context of regional challenges, including persistent gaps in prevention and care, heterogeneous antiretroviral therapy (ART) coverage across countries and populations, limited and uneven scale‐up of biomedical prevention strategies, particularly pre‐exposure prophylaxis (PrEP), delayed HIV diagnosis, socio‐economic disparities, and insufficient investment in healthcare infrastructure and research [2]. Furthermore, political and economic instability in the region exacerbates population mobility, which disrupts continuity of care and undermines prevention strategies, thereby intensifying transmission dynamics [3].

In this context, Uruguay has one of the highest HIV incidence rates in Latin America, with 0.51 new HIV acquisitions per 1000 adults (aged 15–49) in 2023 [4, 5]. Notably, 83% of people diagnosed with HIV are currently receiving ART, underscoring national progress in expanding access and supporting adherence. Furthermore, vertical transmission has consistently remained below 2% in recent years, highlighting sustained prevention success [6]. Despite these advances, while HIV prevalence in the general population is relatively low (0.6%), available national estimates indicate that prevalence exceeds 5% among key populations, including men who have sex with men (MSM), people who inject drugs (PWID), male sex workers, transgender individuals and people in prisons or other closed settings [7]. Thus, a more comprehensive understanding of HIV dynamics among key subpopulations is essential for reducing viral transmission in Uruguay.

Understanding HIV‐1 genetic diversity is essential for detecting prevalent and emerging lineages, documenting how the composition of local epidemics changes over time, and placing national epidemics within broader regional and global patterns [8, 9, 10]. When integrated with epidemiological metadata and phylogenetic tools, HIV sequence data can also support prevention policymaking and public health responses by linking lineage circulation with population‐specific transmission patterns, geographic connectivity, and gaps in prevention and care [11, 12]. This applied value has been demonstrated by routine and national HIV genotyping programmes, where aggregated sequence datasets have been used to refine surveillance priorities, characterize epidemiologically relevant transmission patterns and follow drug‐resistance trends [13, 14, 15].

In Uruguay, previous molecular epidemiological studies indicate a predominance of subtype B and BF1 recombinants, including the circulating recombinant forms (CRFs) 12_BF and 38_BF1 [16, 17, 18], as well as the detection of subtype C [19, 20]. These findings suggest that the HIV epidemic in Uruguay has likely been shaped by cross‐border mobility with Argentina and Brazil [14, 16, 21, 22], facilitated by longstanding historical and cultural ties [23]. Despite these preliminary insights, Uruguay's HIV molecular epidemiology remains insufficiently characterized, and further research is needed to better understand the temporal dynamics and transmission patterns shaping the national epidemic across distinct subpopulations. To address this gap, we conducted the first nationwide, longitudinal molecular epidemiology study of HIV‐1 in Uruguay, using HIV‐1 *pol* sequences and associated metadata from routine genotyping conducted between 2007 and 2021. By characterizing viral lineages, temporal trends and associations with epidemiological metadata, this study provides new insights into HIV‐1 spread within Uruguay and its connection to broader regional dynamics.

## 2 Methods

### 2.1 Routine Genotyping Data

This study screened 1598 partial HIV‐1 *pol* nucleotide sequences generated from samples collected between 2007 and 2021 across all five major Uruguayan regions (Metropolitan Area, Northeast, West, East and Central Area). These included all available sequences from routine genotypic resistance testing performed at the Department of Public Health Laboratories of the Uruguayan Ministry of Public Health (DLSP‐MSP) within the study period. In Uruguay, genotypic resistance testing is indicated in specific clinical contexts, including individuals experiencing virological failure, children with perinatally acquired HIV, pregnant women, cases of recent seroconversion and symptomatic primary HIV infection [24]. Treatment‐history information showed that 64.4% of screened records had at least one previous ART regimen recorded, 28.5% had none and 7.1% had unavailable information.

The sequences spanned the protease and partial reverse transcriptase (PR/RT) regions and were generated by Sanger sequencing following Alemán et al. [25]. Amplicons (∼1100 bp) were bidirectionally sequenced at Macrogen Inc. (Seoul, Republic of Korea). Forward and reverse chromatograms were assembled into consensus sequences at DLSP‐MSP using SeqMan software from the Lasergene package (DNASTAR Inc.); low‐quality ends were trimmed, and ambiguous positions were manually reviewed and coded using IUPAC ambiguity codes when appropriate. Sequences were excluded if they met any of the following criteria: (i) individuals younger than 14 years of age, to minimize potential biases from vertical transmission; (ii) duplicate samples from the same individual, retaining only the earliest available sequence, to avoid redundancy; and/or (iii) failure to meet high‐quality standards as assessed by the Los Alamos HIV Sequence Quality Assessment tool (https://www.hiv.lanl.gov). Low‐quality HIV *pol* sequences (e.g. those with APOBEC‐induced hypermutation or premature stop codons) were excluded because they could bias phylogenetic and recombination inferences [26]. After filtering, 1268 high‐quality HIV *pol* sequences covering the PR/RT regions corresponding to HXB2 positions 2358–3251 were retained for downstream analyses.

### 2.2 Epidemiological Data

Demographic and clinical metadata were extracted from HIV drug‐resistance genotyping request forms completed by treating physicians and submitted to DLSP‐MSP with the corresponding sample. These data were linked to HIV‐1 *pol* sequences using anonymized identifiers and integrated into GENOUY‐HIV, the first national HIV molecular database in Uruguay. We analysed sampling location, gender and reported transmission route. Sampling location was grouped into five macro‐regions: Metropolitan Area, East, Northeast, West and Central Area. Gender was categorized as male, female or transgender, and transmission routes as heterosexual (HTX), MSM and PWID. Sex assigned at birth and gender identity were not recorded separately. Therefore, entries recorded as “transgender” were retained as reported and not further disaggregated. Because MSM and PWID are often stigmatized, underreporting of these exposures and misclassification within the HTX group cannot be excluded. Missing, incomplete or non‐specific entries were coded as unavailable and excluded only from analyses involving the corresponding variable. To contextualize temporal molecular patterns within the national epidemic, annual HIV diagnosis rates and male‐to‐female ratios (M/F) among newly diagnosed people were extracted from national surveillance reports issued by the Uruguayan Ministry of Public Health [6, 7, 27, 28, 29].

### 2.3 HIV‐1 Subtype Classification and Phylogenetic Analysis

HIV‐1 *pol* sequences were initially assigned to subtypes or recombinant forms using three automated tools: REGA v3.0 [30], COMET [31] and RIP [32]. Only concordant results across all tools were considered definitive. Sequences with discordant or unassigned outputs were further screened for mosaic structure using Bootscan implemented in the SimPlot package v3.5.1 [33] (300‐nt sliding window, 10‐nt step size). Mosaics consistent with known CRFs were annotated accordingly, while others were retained for further analyses. To confirm lineage assignments, three targeted phylogenetic validations were performed: (i) sequences classified as subtypes/sub‐subtypes were co‐analysed with subtype‐specific reference panels; (ii) sequences assigned to CRFs were evaluated against CRF‐specific reference datasets; and (iii) recombinant sequences not attributable to any known CRF were analysed with a comprehensive panel including reference sequences from all recognized CRFs. Reference panels were derived from the Los Alamos HIV Sequence Database 2020 Subtype Reference Alignment (ID 120RG1). Alignments were generated using MAFFT [34]. To minimize the influence of convergent evolution driven by ART selective pressure, we excluded a conservative set of codons associated with clinically relevant ART drug resistance in PR (30, 32, 46, 47, 48, 50, 54, 76, 82, 84, 88 and 90) and RT (41, 65, 67, 69, 70, 74, 100, 101, 103, 106, 115, 138, 151, 181, 184, 188, 190, 210, 215, 219 and 230) [35]. Phylogenetic trees were inferred using IQ‐TREE v1.6.12 [36], with the best‐fit substitution model selected by ModelFinder [37]. Node support was assessed using the SH‐aLRT test [38], with values ≥0.80 considered strong nodal support.

### 2.4 Statistical Analysis

HIV‐1 variant frequencies were estimated overall and by macro‐region. Univariate associations between major HIV‐1 variants (>40 sequences) and macro‐region, gender or reported transmission route were assessed by comparing, for each variant, the distribution of epidemiological categories among sequences assigned to that variant with that among all other variants combined. The aggregated comparison group was used as the empirical background distribution of the genotyped dataset and was not interpreted as a biologically homogeneous lineage group. Fisher's exact tests were used to assess overall associations, and Z‐tests for proportions were used to identify category‐level differences (two‐sided, α = 0.05), using IBM SPSS Statistics v22.0.

### 2.5 Ethical Aspects

This work was approved by the Ethics Committee of the Centro Universitario Regional (CENUR) Litoral Norte of the Universidad de la República (UdelaR), Uruguay, under Registration No. 311170‐500476‐21. Data were compiled in 2022 and analysed between 2022 and 2025. All methods were performed in accordance with the relevant guidelines and regulations.

## 3 Results

### 3.1 Molecular Profile of HIV‐1 Lineages

A total of 1268 HIV‐1 *pol* sequences from samples collected between 2007 and 2021 were analysed to characterize molecular diversity in Uruguay. Based on an average of nearly 900 new HIV diagnoses per year in Uruguay during this period [6], these sequences correspond to approximately 9.4% of all reported HIV diagnoses. The sampling period encompassed distinct phases of the Uruguayan HIV epidemic: national HIV diagnosis rates increased significantly from 17.3 cases per 100,000 inhabitants in 2007 to 34.7 in 2012 (*p* = 0.0032), declined significantly to 23.2 in 2017 (*p* = 0.0002) and then remained broadly stable through 2024 (Figure S1).

**FIGURE 1:**
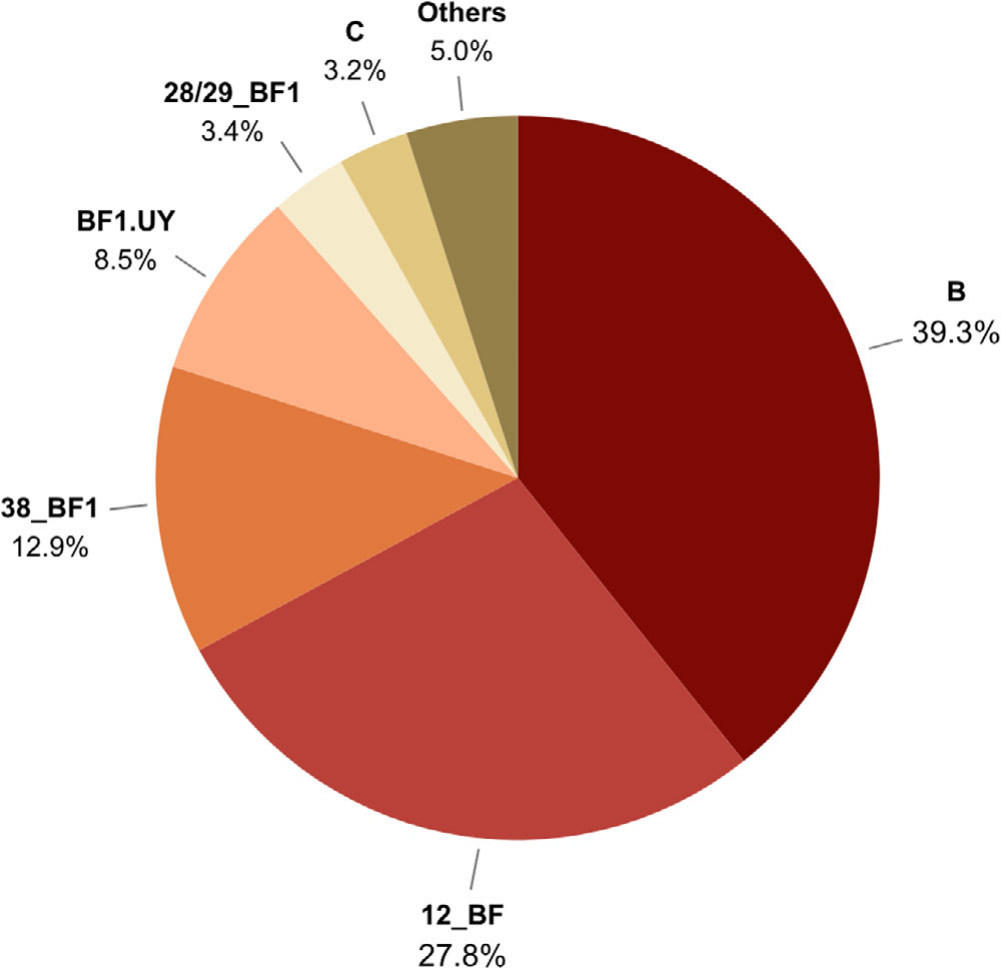
Relative frequency of HIV‐1 lineages in the Uruguayan genotyped dataset, 2007–2021. The chart shows the proportion of HIV‐1 *pol* sequences assigned to each major lineage. The “Others” category includes lineages that individually represented ≤1.5% of the dataset (46_BF1, 31_BC, F1, 17_BF1, 20_BG, 24_BG, 01_AE, 19_cpx, 60_BC, 41_CD and A1). See Table S1 for counts and detailed frequencies.

Automated subtyping, supported by phylogenetic validation against subtype‐ and CRF‐specific reference datasets, assigned 91.5% (*n* = 1160) of sequences to sixteen recognized HIV‐1 lineages: four subtypes/sub‐subtypes (B, C, A1 and F1) and twelve CRFs (01_AE, 12_BF, 17_BF1, 19_cpx, 20_BG, 24_BG, 28/29_BF1, 31_BC, 38_BF1, 41_CD, 46_BF1 and 60_BC) (Figure 1). Subtype B was the predominant lineage (39.3%), followed by CRFs 12_BF (27.8%), 38_BF1 (12.9%), 28/29_BF1 (3.4%) and subtype C (3.2%), while the remaining subtypes and recombinant forms together represented 5.0% of the dataset (Figures 1 and S2; Table S1).

**FIGURE 2:**
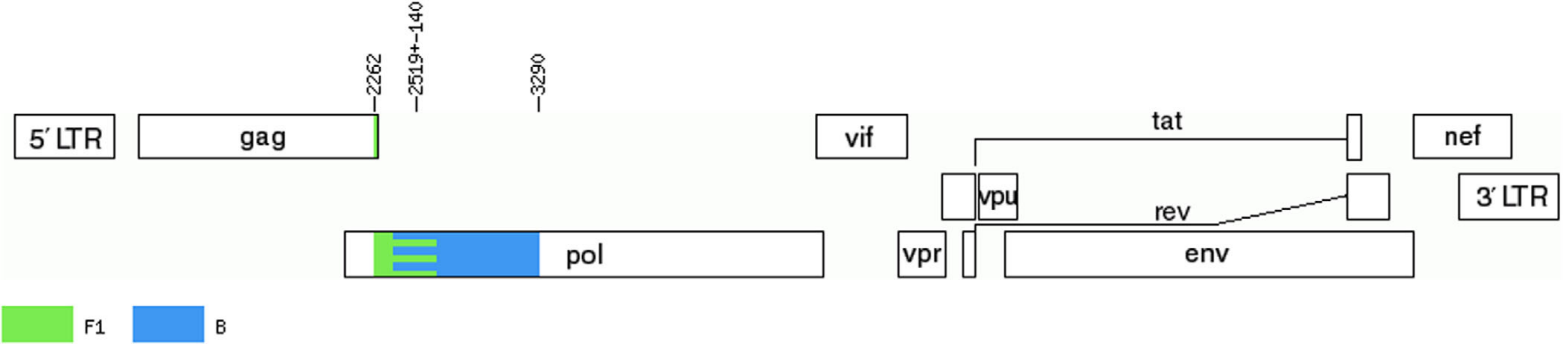
Schematic representation of the recombination pattern inferred for BF1.UY sequences within the analysed *pol* fragment. The analysed *pol* region is shown with subtype assignments indicated in green and blue, corresponding to sub‐subtype F1 and subtype B, respectively. Vertical markers indicate the range of breakpoint positions estimated across the 108 BF1.UY sequences, using HXB2 reference numbering. HIV‐1 genomic landmarks are shown for orientation.

The remaining 108 *pol* sequences (8.5%) were not assignable to any recognized lineage after automated subtyping and phylogenetic comparison with reference sequences. Recombination analysis revealed a consistent mosaic pattern with a breakpoint within HXB2 positions 2400–2600, delineating an upstream F1‐derived fragment and a downstream B‐derived fragment (Figure 2). In the inferred maximum‐likelihood phylogeny, these B/F1 recombinants formed a strongly supported clade (SH‐aLRT = 0.97), provisionally designated BF1.UY, which branched from a basal node as part of a well‐supported polytomy (SH‐aLRT = 0.91) with two additional lineages: one comprising 38_BF1 and 42_BF1 reference strains (SH‐aLRT = 0.80), and another consisting of 17_BF1 (SH‐aLRT = 0.96) (Figure S3).

### 3.2 Geographic Distribution of HIV‐1 Lineages in Uruguay

**FIGURE 3:**
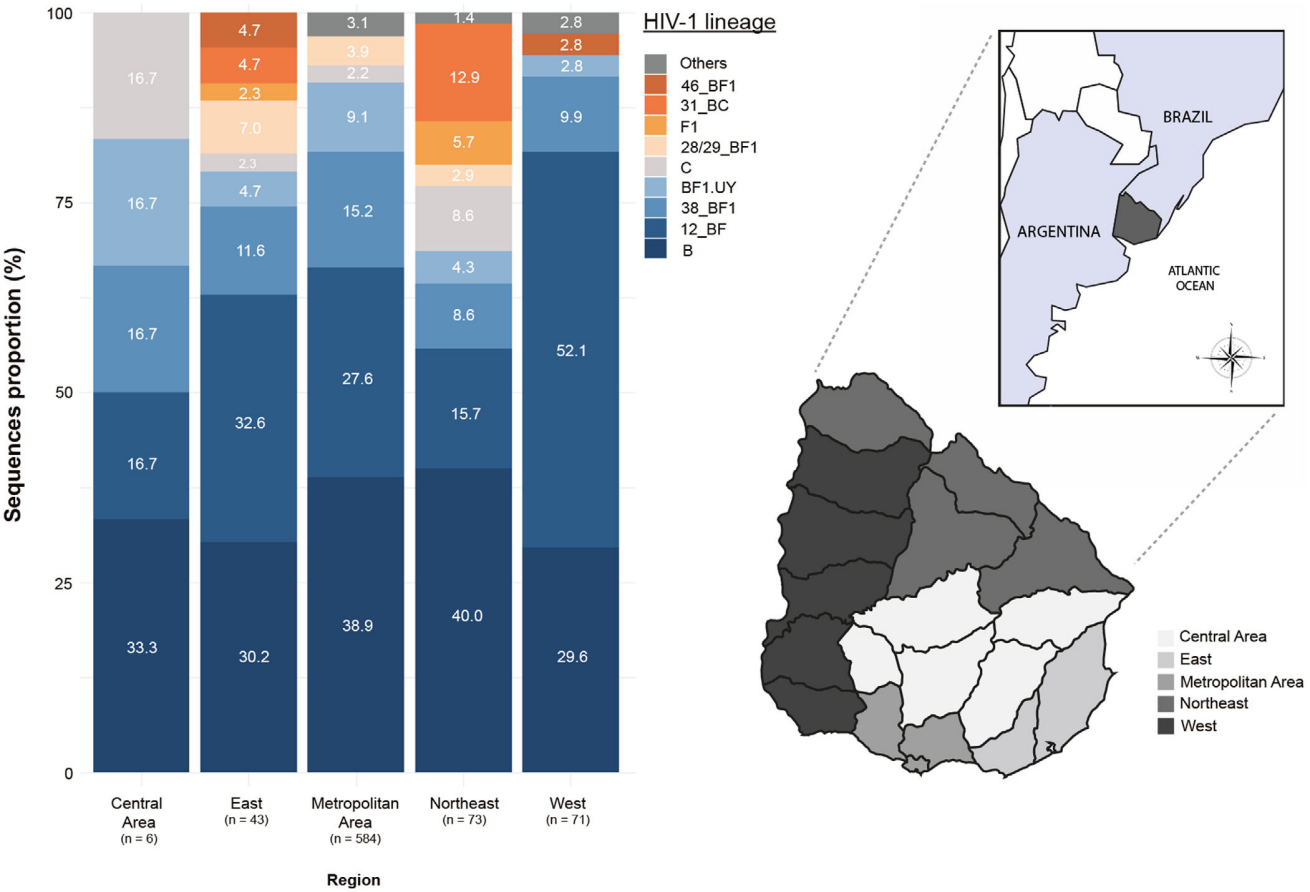
Geographic distribution of HIV‐1 lineages in the Uruguayan genotyped dataset. Stacked bars show lineage proportions among sequences with available sampling‐location metadata, stratified by macro‐region; numbers below each bar indicate the number of sequences analysed per region. Major lineages are shown in shades of blue, lower‐frequency lineages that reached ≥2.0% in at least one macro‐region are shown in orange and all remaining lineages are grouped as “Others” in grey. The map shows the Uruguayan macro‐regions used in the analysis and their geographic context relative to Argentina and Brazil.

Of the 1268 *pol* sequences analysed, 777 (61.3%) contained sampling‐location metadata. Most originated from the Metropolitan Area (75.2%), followed by the Northeast (9.4%), West (9.1%), East (5.5%) and Central Area (0.8%), a geographic distribution that roughly mirrors that of newly diagnosed HIV cases in the country [6, 7, 27, 28, 29]. Regional lineage frequencies revealed both widespread dissemination of the major lineages and marked heterogeneity in border‐associated variants (Figure 3). Subtype B occurred at relatively uniform frequencies across macro‐regions (≈30%–40%) and predominated in the Metropolitan, Northeast and Central regions. 38_BF1 and BF1.UY also occurred countrywide at relatively uniform moderate (8%–16%) and low (<10%) frequencies, respectively. In contrast, 12_BF displayed marked geographic heterogeneity, being most prevalent in the East (32.6%) and West (52.1%), but less frequent in the Northeast (∼16%). Subtype C and 31_BC also displayed heterogeneous distribution, being mainly restricted to the Northeast and East. Pairwise Z‐tests indicated that the West, which borders Argentina, had a higher proportion of 12_BF and a lower proportion of subtype B than both the Northeast and Metropolitan regions; whereas the Northeast, which borders Brazil, had a higher proportion of subtype C and lower proportions of 12_BF and 38_BF1 than the Metropolitan Area (Figure 3).

### 3.3 Association Between HIV‐1 Lineages and Gender

**FIGURE 4:**
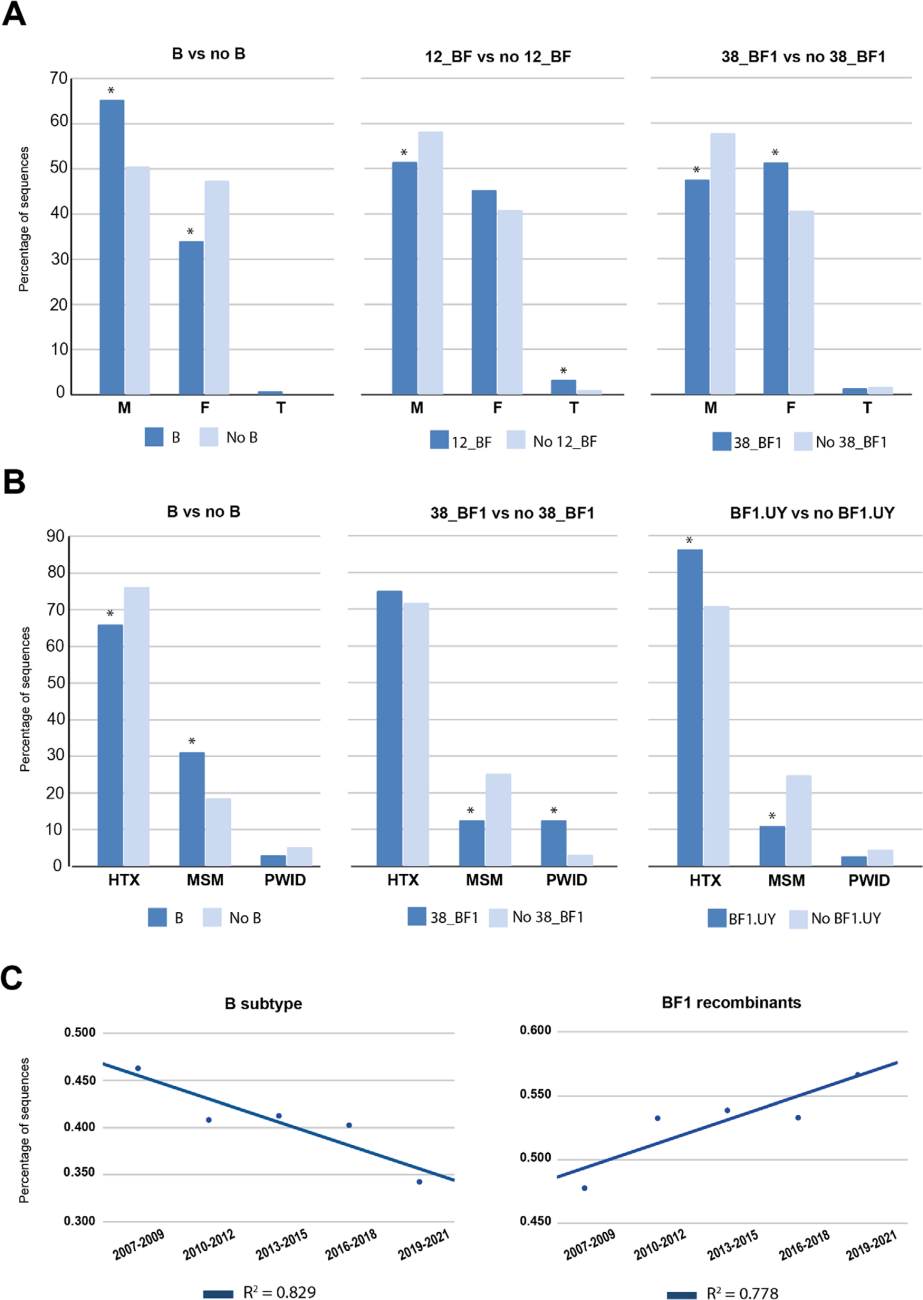
Associations between HIV‐1 lineages and gender, transmission route, and sampling period. Analyses were restricted to lineages represented by >40 sequences. (A) Gender distribution for lineages significantly associated with gender by Fisher's exact test. (B) Transmission‐route distribution for lineages significantly associated with transmission route by Fisher's exact test. In panels A and B, each lineage is compared with all remaining lineages combined; bars represent the percentage of sequences in each category, and asterisks indicate significant differences in Z‐tests for proportions (*p* < 0.05). (C) Temporal trends in the relative frequency of subtype B and BF1 recombinants from 2007 to 2021, summarized in non‐overlapping 3‐year periods. BF1 recombinants include 12_BF, 17_BF1, 28/29_BF1, 38_BF1, 46_BF1 and BF1.UY. Lines represent linear regressions of triennial proportions, with *R* ^2^ values shown for each group. Abbreviations: F, female; HTX, heterosexual transmission; M, male; MSM, men who have sex with men; PWID, people who inject drugs; T, transgender.

Gender information was available for 1230 sequences (97.0% of the dataset). Most Uruguayan HIV‐1 *pol* sequences were from men (56.4%), followed by women (42.0%), and people recorded as transgender (1.6%). The gender composition of the genotyped dataset changed over time. Annual comparison with national surveillance data showed that the M/F ratio in the genotyped dataset fluctuated over time and did not consistently mirror the ratio observed among newly diagnosed HIV cases, particularly after 2010, when the genotyped dataset showed a lower M/F ratio than the national epidemic (Figure S1). Fisher's exact test revealed significant associations between gender and subtype B (*p* < 0.001), 12_BF (*p* = 0.005) and 38_BF1 (*p* = 0.036). Z‐tests for proportions showed that, relative to non‐B variants, subtype B was overrepresented among men (65.2% vs. 50.5%) and underrepresented among women (34.0% vs. 47.3%). In contrast, 12_BF was relatively enriched among people recorded as transgender (3.3% vs. 1.0%) and depleted among men (51.5% vs. 58.2%) compared with non‐12_BF variants; whereas 38_BF1 was enriched among women (51.3% vs. 40.6%) and depleted among men (47.5% vs. 57.7%) compared with non‐38_BF1 variants (Figure 4A and Table S2).

### 3.4 Associations Between HIV‐1 Lineages and Transmission Route

Transmission route data were available for 854 sequences (67.4% of the dataset). HIV‐1 *pol* sequences were obtained from individuals in three transmission groups: HTX (72.1%), MSM (23.5%) and PWID (4.3%). Fisher's exact tests revealed significant associations between transmission route and subtype B (*p* < 0.001), 38_BF1 (*p* < 0.001) and the BF1.UY clade (*p* = 0.013). No statistically significant association was detected between transmission route and 12_BF, although most 12_BF sequences with available transmission‐route data were reported as HTX. Z‐tests for proportions indicated that, relative to non‐B variants, subtype B was more frequently associated with MSM transmission (31.1% vs. 18.6%). In contrast, 38_BF1 showed a higher proportion of PWID transmission (12.5% vs. 3.1%) and a lower proportion of MSM transmission (12.5% vs. 25.2%) compared to non‐38_BF1 variants; while the BF1.UY clade was predominantly associated with HTX transmission (86.3% vs. 70.8%) compared to non‐BF1.UY variants (Figure 4B and Table S3).

### 3.5 Temporal Trends in Major HIV‐1 Lineages

Temporal patterns were evaluated in non‐overlapping 3‐year periods (2007–2009, 2010–2012, 2013–2015, 2016–2018, 2019–2021) for the six most frequent lineages and for a composite group of all BF1 recombinants (12_BF, 17_BF1, 28/29_BF1, 38_BF1, 46_BF1 and BF1.UY). Pearson's correlation on triennial proportions showed a significant negative temporal trend for subtype B (*R* ^2^ = 0.829; *p* = 0.033) and a positive temporal trend for the BF1 recombinant group (*R* ^2^ = 0.778; *p* = 0.0479) (Figure 4C). These opposite patterns indicate an increasing relative contribution of BF1 recombinants within the genotyped dataset, but should be interpreted in light of substantial changes in national HIV diagnosis rates and temporal differences between the M/F ratio of the genotyped dataset and that observed in national surveillance data (Figure S1).

## 4 Discussion

This study provides the most comprehensive longitudinal characterization of HIV‐1 molecular diversity in Uruguay to date, revealing a dynamic, heterogeneous epidemic dominated by subtype B and BF1 recombinant forms. Among genotyped individuals, subtype B remained the most frequent lineage (39.3%), whereas BF1 recombinant forms, considered collectively, constituted the predominant genetic component (55%). The high diversity and prevalence of BF1 forms underscore that Uruguay harbours a recombination‐driven epidemic landscape, with multiple related BF1 lineages undergoing local diversification. This pattern is consistent with the broader regional diversification of BF1 recombinants reported in Argentina [39, 40, 41] and Brazil [14, 20].

The 2007–2021 sampling interval analysed here captures a period of substantial epidemiological change in Uruguay, including an increase in HIV diagnosis rates up to 2012, a subsequent decline through 2017, and a period of broad stability that extended through 2024. Against this background, subtype B declined, and BF1 recombinants increased within the genotyped dataset, with the B/BF1 ratio decreasing from 1.02 in 2007–2009 to 0.62 in 2019–2021. Because these estimates derive from a selected genotyped population rather than systematic population‐level molecular surveillance, they should be interpreted with caution. Notably, from 2011 onwards, the M/F ratio among genotyped individuals was generally lower than that observed among newly diagnosed cases nationally, suggesting a relative enrichment of women in the genotyped dataset compared with the national epidemic. Given the lineage–gender associations observed in this study, this gender imbalance in the genotyped dataset may have contributed to the apparent decline in subtype B and increase in BF1 recombinants. Thus, the temporal trends described here should be interpreted primarily as patterns within the genotyped population, rather than as direct estimates of lineage replacement in the national epidemic. However, the available data preclude precise disentangling of the relative contributions of sampling composition and changes in circulating lineage diversity.

The Uruguayan epidemic shows limited geographic structuring, suggesting frequent viral mixing among geographic regions. Spatial signals were largely confined to border regions with neighbouring countries. 12_BF, originally described in Argentina and Uruguay [42], is particularly frequent in the West and along the eastern Atlantic coast, consistent with intense cross‐border population exchange with Argentina and seasonal tourism‐related flows [23, 43]. By contrast, lineages associated with the Brazilian epidemic, including 28/29_BF1, 31_BC, subtype C and sub‐subtype F1 [14], are mainly concentrated in the East and Northeast, where cross‐border mobility with southern Brazil is greatest [44, 45]. Subtype B and the other major BF1 recombinant lineages (38_BF1 and BF1.UY), in turn, showed roughly similar frequencies across all macro‐regions, consistent with their long‐standing dissemination at the national scale. Taken together, these spatial patterns indicate that Uruguay's current HIV‐1 diversity reflects the combined effects of early nationwide dissemination of subtype B, multiple introductions of non‐B variants from neighbouring countries and successful amplification of a few local recombinant BF1 lineages.

Major HIV‐1 lineages circulating in Uruguay display distinct demographic and transmission‐route profiles in univariate analyses. Subtype B is overrepresented among men and MSM, consistent with multiple international studies describing the historical spread of this subtype within MSM transmission networks [14, 46, 47, 48]. In contrast, 38_BF1 shows higher representation among women and PWID compared with other lineages, in agreement with findings reported in the original description of this CRF [49]. The persistence of these epidemiological associations over more than two decades is consistent with the continued circulation of 38_BF1 within population groups historically affected by parenteral exposure. Although 12_BF showed no statistically significant association with any transmission category, most (74%) of the analysed 12_BF sequences were linked to HTX, consistent with observations from Argentina showing a preferential association of this CRF with heterosexual exposure [50, 51]. Notably, 12_BF was also the most common variant (55%) among people recorded as transgender in our dataset, suggesting disproportionate representation of this lineage in this group, a pattern that, to our knowledge, has not been reported in other regional studies of 12_BF. However, the small number of people recorded as transgender and incomplete recording of sex assigned at birth and gender identity preclude further inference about transmission dynamics in this population.

Within the BF1‐enriched background, the BF1.UY clade identified in this study was one of the most distinctive findings in the Uruguayan dataset. Phylogenetic and recombination analyses indicate that BF1.UY is best interpreted as a family of closely related BF1 recombinants rather than a single CRF. The BF1.UY clade is predominantly associated with reported HTX transmission, consistent with the prominent role of heterosexual networks in the regional dissemination of BF1 recombinants [42, 51]. However, our analyses were restricted to partial *pol* sequences and focused on recognized CRF references, without fully representing the diversity of URF_BF1 recombinants from neighbouring countries. Therefore, the BF1.UY clade should be interpreted as a provisional *pol*‐defined BF1 recombinant lineage identified within the Uruguayan dataset. Broader regional sampling and near‐full‐length genome sequencing will be required to determine whether the BF1.UY clade represents a locally diversified constellation of related URFs, one or more yet‐undescribed local CRFs, or part of the wider spectrum of South American URF_BF1 and/or CRF_BF1 lineages.

These results should be interpreted in light of several limitations. First, the GENOUY‐HIV dataset comprises a convenience sample of individuals for whom genotypic resistance testing was requested and was enriched for individuals with evidence of prior ART exposure; therefore, potential sampling bias may limit the representativeness of lineage distribution estimates. Because sequences generated in the context of treatment monitoring or virological failure may reflect HIV acquisitions that occurred several years before sampling, the lineage frequencies and temporal patterns reported here likely capture historical viral diversity more closely than current transmission dynamics. Second, analyses were limited to the PR/RT region, which is routinely generated through drug‐resistance testing and can inform initial lineage assignment [8], but does not allow full resolution of the genomic mosaic structure, and may, therefore, underestimate HIV‐1 genetic diversity and hinder the clear identification of new or established CRFs. Third, for a substantial proportion of individuals (∼30%–40%), information on sampling location and transmission route was missing, potentially limiting the resolution of lineage‐level associations with population subgroups. Despite these limitations, our genotyped dataset comprised 9.4% of all reported HIV diagnoses in Uruguay during the study period, a proportion at the upper end of the estimated coverage of comparable national HIV‐1 molecular epidemiology surveys in Latin America [14, 52, 53, 54, 55, 56], and provides an important molecular epidemiological perspective on a period of significant epidemic shifts in Uruguay.

## 5 Conclusions

The integration of clinical genotyping, phylogenetic analysis and epidemiological metadata provides the first nationwide longitudinal view of HIV‐1 molecular diversity in Uruguay, showing that this diversity is largely shaped by the sustained circulation and local diversification of subtype B and BF1 recombinant lineages over the 2007–2021 period. The BF1 recombinant group exhibited a growing contribution throughout the study period within the genotyped dataset, consistent with changes in the composition of the population undergoing routine resistance genotyping, changes in circulating viral diversity or both. Although this temporal pattern should be interpreted cautiously, it identifies BF1 recombinants as a major and dynamic component of HIV‐1 molecular diversity in Uruguay that requires continued monitoring. Subtype B and three major BF1 recombinants (12_BF, 38_BF1 and BF1.UY) were detected in all five macro‐regions, although their frequencies varied according to geographic location, gender and transmission‐route category. Such associations are consistent with partially overlapping HIV transmission networks among subpopulations in Uruguay, underscoring the potential value of tailored prevention and surveillance strategies. Furthermore, diversity patterns in Uruguayan border regions likely reflect intense connectivity with Argentina and Brazil, highlighting the importance of integrated molecular surveillance systems that combine sequence data, epidemiological metadata and regional collaboration to monitor cross‐border HIV transmission dynamics and emerging recombinant lineages, thereby supporting coordinated prevention strategies.

## Author Contributions

*Conceived the study*: DM. *Refined the study design and analytical strategy*: DM, GB and AMS. *Generated and curated the genotyping data*: RF, DR, MB, MNC and HC. *Provided clinical input*: RF and SC. *Performed the analyses*: AAP. *Interpreted the data*: AAP, DM, GB, AMS, AL and SC. *Drafted the first version of the manuscript*: AAP and DM. *Critically revised the manuscript for important intellectual content*: GB, AMS and AL. All authors read and approved the final manuscript and agree to be accountable for all aspects of the work.

## Funding

This work was supported by the Comisión Sectorial de Investigación Científica (CSIC–Uruguay) (CSIC_VUSP_Modalidad‐2‐2021), by the Fondo Carlos Vaz Ferreira (FVF) from the Dirección Nacional de Innovación, Ciencia y Tecnología (DICYT), Ministry of Education and Culture (MEC), Uruguay (Project FVF_2023_482), and by the L'Oréal Uruguay For Women in Science Fellowship Program. Agustina A. Podchibiakin is the recipient of a predoctoral scholarship (POS_NAC_2023_1_178397) from ANII (Uruguay).

## Conflicts of Interest

The authors have no conflicts of interest.

## Supporting information




**Figure S1**: Temporal context of the Uruguayan HIV epidemic and gender composition of the genotyped dataset.

**Figure S2**: Maximum‐likelihood phylogenies of HIV‐1 lineages circulating in Uruguay.

**Figure S3**: Maximum‐likelihood phylogeny of unassigned B/F1 recombinant HIV‐1 *pol* sequences.

**Table S1**: Distribution of HIV‐1 lineages in the Uruguayan genotyped dataset, 2007–2021.
**Table S2**: Association between HIV‐1 lineages and gender.
**Table S3**: Association between HIV‐1 lineages and transmission route.

## Data Availability

All HIV‐1 *pol* sequences analysed in this study have been deposited in GenBank under accession numbers PZ351606–PZ351641 and PZ351643–PZ352874.
