# Supplementary material for: Nationwide Molecular Epidemiology of HIV‐1 in Uruguay (2007–2021): Lineage Diversity, BF1 Recombinant Complexity and Epidemiological Patterns
Source: J Int AIDS Soc. 2026 Jul 25;29(Suppl 3):e70157. doi: 10.1002/jia2.70157 (PMC13401711; doi:10.1002/jia2.70157)
Supplement: Supplementary file 1 — Figure S1: Temporal context of the Uruguayan HIV epidemic and gender composition of the genotyped dataset. [file JIA2-29-e70157-s003.docx]

**
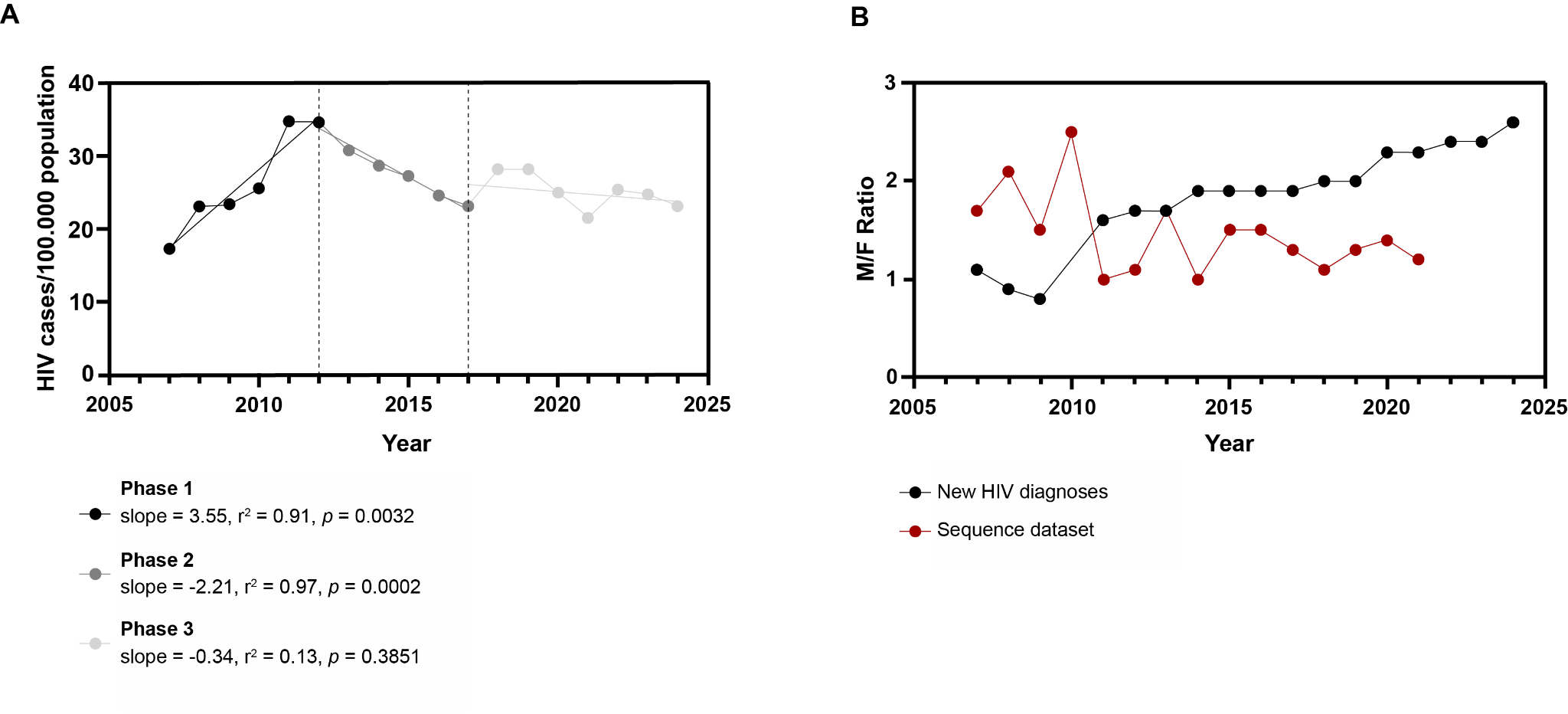
 Supplementary Figure 1. Temporal context of the Uruguayan HIV epidemic and gender composition of the genotyped dataset.** (A) Annual HIV diagnosis rates per 100,000 population in Uruguay between 2007 and 2024. Dotted vertical lines indicate three epidemiological phases: increase up to 2012, decline through 2017, and broad stability thereafter. Slopes, R² values and p-values are shown for each phase. (B) Male-to-female ratio among newly diagnosed HIV cases nationally and among individuals included in the genotyped sequence dataset.
