# Supplementary material for: Nationwide Molecular Epidemiology of HIV‐1 in Uruguay (2007–2021): Lineage Diversity, BF1 Recombinant Complexity and Epidemiological Patterns
Source: J Int AIDS Soc. 2026 Jul 25;29(Suppl 3):e70157. doi: 10.1002/jia2.70157 (PMC13401711; doi:10.1002/jia2.70157)
Supplement: Supplementary file 2 — Figure S2: Maximum‐likelihood phylogenies of HIV‐1 lineages circulating in Uruguay. [file JIA2-29-e70157-s001.docx]

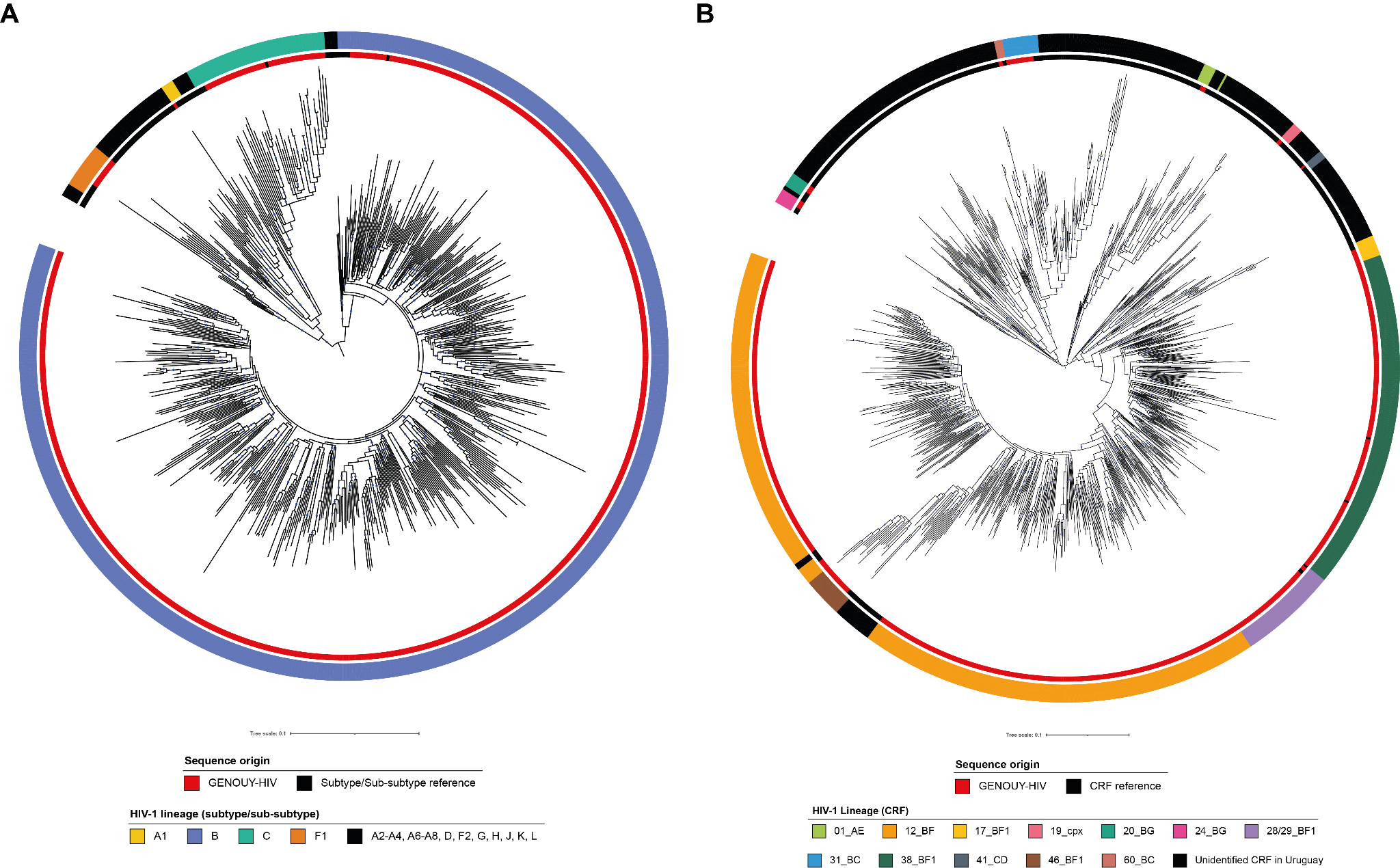


**Supplementary Figure 2. Maximum-likelihood phylogenies of HIV-1 lineages circulating in Uruguay. (A) Subtypes and sub-subtypes tree.** Based on 605 sequences, 549 from the GENOUY-HIV database and 56 reference sequences representing all pure HIV-1 subtypes and sub-subtypes. **(B) CRFs tree.** Based on 967 sequences, 611 from the GENOUY-HIV database and 356 reference sequences representing all described CRFs. In both trees, the inner ring indicates sequences from the GENOUY-HIV database (red) and reference sequences (black). The outer ring shows the different lineages identified (color legend). The tree was midpoint-rooted. All nodes marked with circles have SH-aLRT support ≥ 0.80. Branch lengths are scaled to nucleotide substitutions per site. Reference sequences were obtained from the Los Alamos HIV Database.
