# Supplementary material for: Nationwide Molecular Epidemiology of HIV‐1 in Uruguay (2007–2021): Lineage Diversity, BF1 Recombinant Complexity and Epidemiological Patterns
Source: J Int AIDS Soc. 2026 Jul 25;29(Suppl 3):e70157. doi: 10.1002/jia2.70157 (PMC13401711; doi:10.1002/jia2.70157)
Supplement: Supplementary file 3 — Figure S3: Maximum‐likelihood phylogeny of unassigned B/F1 recombinant HIV‐1 pol sequences. [file JIA2-29-e70157-s004.docx]

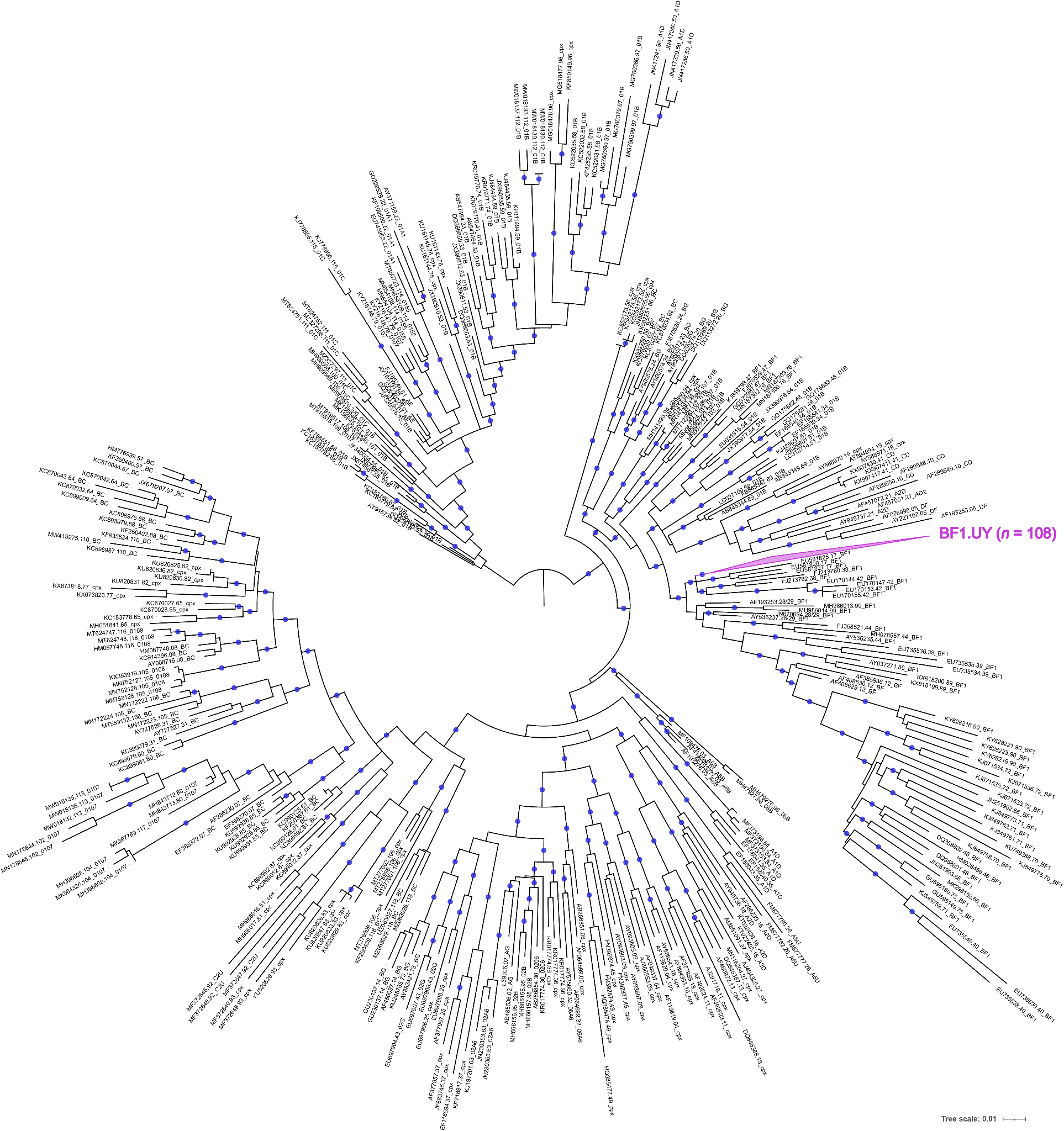


**Supplementary Figure 3. Maximum-likelihood phylogeny of unassigned B/F1 recombinant HIV-1 *pol* sequences.** The tree includes 108 GENOUY-HIV B/F1 recombinant sequences and 356 reference sequences representing recognized HIV-1 circulating recombinant forms (CRFs) retrieved from the Los Alamos HIV Database. The BF1.UY cluster is highlighted in pink. Blue circles indicate nodes with SH-aLRT support ≥0.80. Branch lengths are proportional to nucleotide substitutions per site. The tree was midpoint-rooted. CRF, circulating recombinant form; SH-aLRT, Shimodaira-Hasegawa approximate likelihood ratio test.
