# Supplementary material for: Nationwide Molecular Epidemiology of HIV‐1 in Uruguay (2007–2021): Lineage Diversity, BF1 Recombinant Complexity and Epidemiological Patterns
Source: J Int AIDS Soc. 2026 Jul 25;29(Suppl 3):e70157. doi: 10.1002/jia2.70157 (PMC13401711; doi:10.1002/jia2.70157)

**Supplementary Table 1.**

**Distribution of HIV-1 lineages in the Uruguayan genotyped dataset, 2007-2021.**


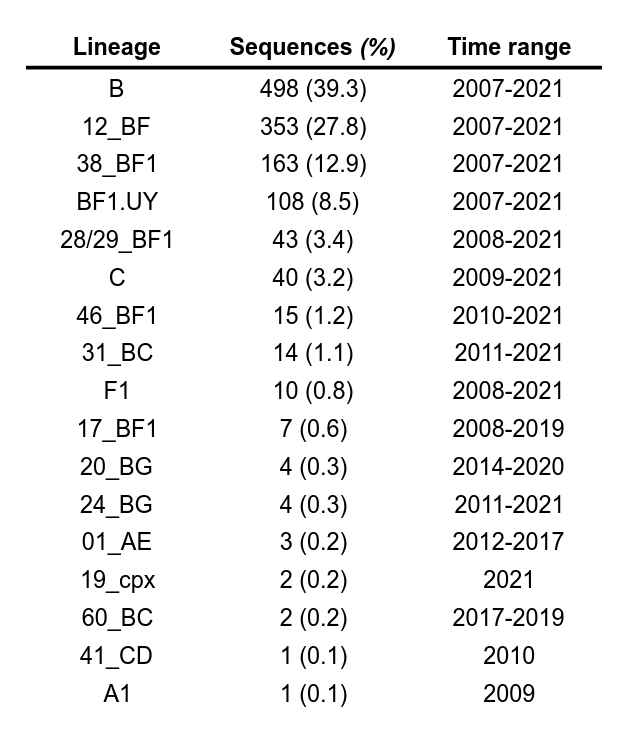


**Supplementary Table 2. Association between HIV-1 lineages and gender. Comparisons between each lineage and all remaining lineages combined were performed using Fisher’s exact test. Significant p-values are shown in bold. M: male; F: female; T: transgender.**


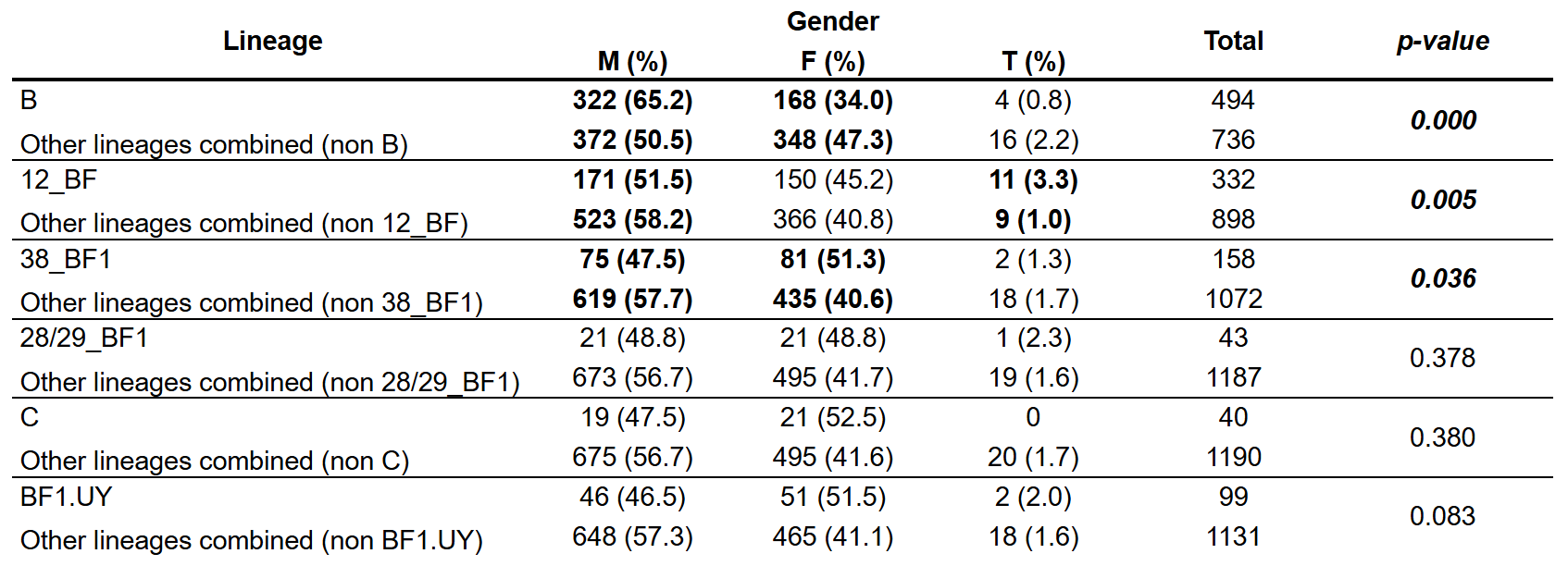


**Supplementary Table 3. Association between HIV-1 lineages and transmission route. Comparisons between each lineage and all remaining lineages combined were performed using Fisher’s exact test. HTX: heterosexual; MSM: men who have sex with men; PWID: people who inject drugs.**


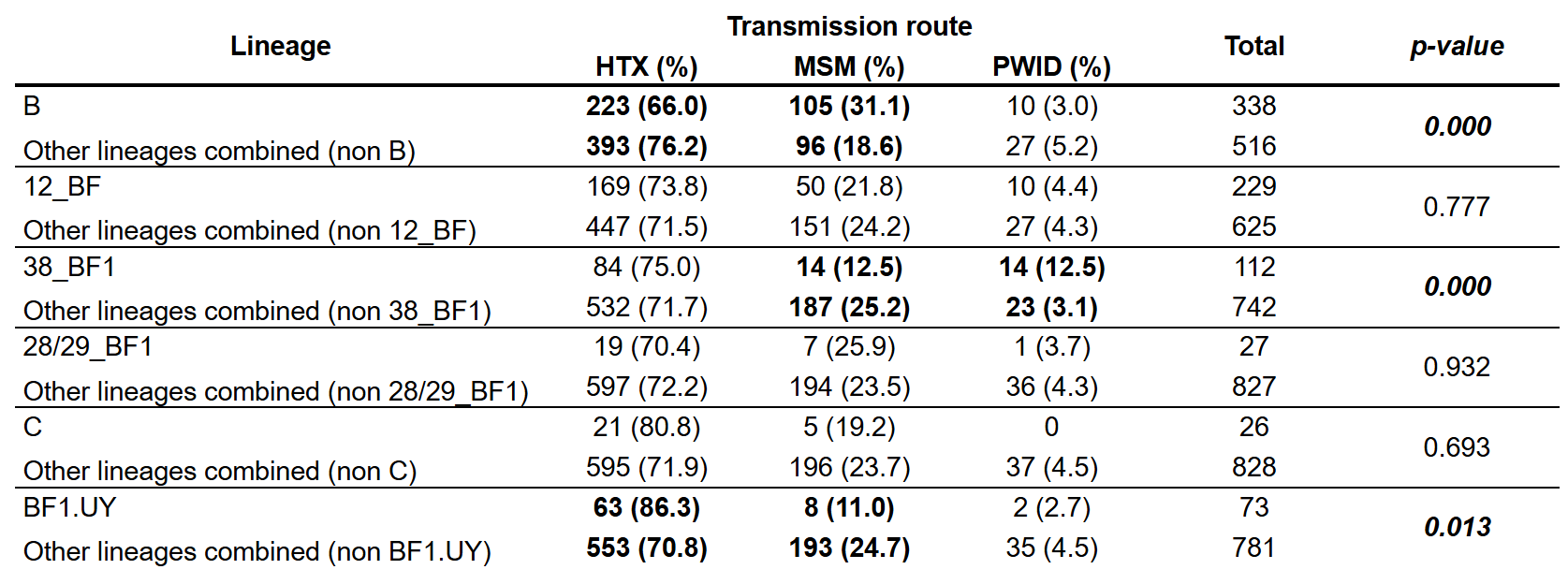

Supplement: Supplementary file 4 — Table S1: Distribution of HIV‐1 lineages in the Uruguayan genotyped dataset, 2007–2021. Table S2: Association between HIV‐1 lineages and gender. Table S3: Association between HIV‐1 lineages and transmission route. [file JIA2-29-e70157-s002.docx]
